# Indigo-Uria

**Published:** 1907-11-02

**Authors:** 


### INDIGO-URIA.

A woman, aged 31, had suffered a miscarriage at the second month, followed by severe sepsis, with great oscillations on her temperature chart. Suitable treatment had saved her life, and she was much better except for moderate cystitis. It had been for some time noted that her urine, which was alkaline in reaction, and pale yellow when passed, spontaneously darkened on standing—becoming first yellowish-green, then deep greenish-brown. The change in colour occurred simultaneously with fermentative changes in the urine, and the condition persisted for nine weeks, until lavage of the bladder with a solution of silver nitrate was resorted to. The latter cured the cystitis, and at the same time the urine ceased to go dark on standing exposed to the air.

Many different abnormal conditions of the urine darken in this way—for example, melanuria, carboluria, and alkaptonuria. The above case, however, was none of these, but indigo-uria.

The darkening began at the top of the specimen as it stood, and extended gradually down the contents of the glass, the surface always being darker than the layers below; on certain days it was possible to see numerous fine blue particles on the top of the urine.

Filtration and centrifugalisation rendered the specimen less dark in colour. Amongst the substances left upon the filter paper were minute crystals of indigotine, and similar crystals, along with pus cells, and so forth, were present in a drop of the centrifugalised deposit. Shaking the urine up with chloroform led to extraction of the indigotine by the latter, the chloroform consequently assuming a bluish violet colour.

Indigo-uria is not extremely rare. It may be produced experimentally by giving a sufficient dose of indol by the mouth. It occurs spontaneously in many urines that are alkaline and decomposing. It is particularly to be met with in the elderly, and it often coincides with cystitis, pyelitis, pyelonephritis, or a bad general state of health. It is most clearly seen when the urine, rich in indoxyl, excretes the latter in combination with glycuronic acid instead of with sulphuric acid. Indican is potassium indoxylsulphate, and is less easily decomposed by bacteria in an alkaline medium than are the indoyl-glycuronates; it is decomposition of the latter which gives rise to the spontaneous darkening of the urine by liberation of indigotine, which is the cause of indigo-uria.

Indigo-uria is usually transient; in the present case it lasted longer than is usual, but it disappeared directly the silver nitrate treatment was adopted and the cystitis cured.

